# A14 INVESTIGATING THE IMPACT OF PARKINSON’S DISEASE-ASSOCIATED GENE *PINK1* ON INTESTINAL HOMEOSTASIS AND RESPONSE TO INFECTION

**DOI:** 10.1093/jcag/gwae059.014

**Published:** 2025-02-10

**Authors:** J Pei, S J Recinto, A Kazanova, L Burns, A MacDonald, C Gavino, M Desjardins, J A Stratton, S Gruenheid

**Affiliations:** Microbiology and Immunology, McGill University, Montreal, QC, Canada; Microbiology and Immunology, McGill University, Montreal, QC, Canada; Microbiology and Immunology, McGill University, Montreal, QC, Canada; Microbiology and Immunology, McGill University, Montreal, QC, Canada; Microbiology and Immunology, McGill University, Montreal, QC, Canada; Microbiology and Immunology, McGill University, Montreal, QC, Canada; Universite de Montreal, Montreal, QC, Canada; Microbiology and Immunology, McGill University, Montreal, QC, Canada; Microbiology and Immunology, McGill University, Montreal, QC, Canada

## Abstract

**Background:**

Intestinal epithelial cells (IECs) provide an essential physical barrier between luminal contents and host tissue. Dysregulation of IECs leads to barrier dysfunction, causing pathologies in both intestinal and extra-intestinal diseases. While Parkinson’s Disease (PD) is primarily a neurodegenerative disorder, increasing evidence links PD progression and gastrointestinal dysfunction. Our group developed a model to investigate the role of the gut in PD, demonstrating that mice with genetic ablation of the PD-associated gene *Pink1* exhibited motor phenotypes only when previously infected with gram-negative *Citrobacter rodentium* intestinal bacteria. As *Pink1* is expressed in IECs and the colonic lamina propria, we hypothesize that PD-associated gene mutations directly affect the epithelium and impact early PD pathophysiology.

**Aims:**

1. To characterize the transcriptional profiles of *Pink1* WT and KO IECs at baseline and following *in vivo C. rodentium* infection.

2. Use *in vitro* cell line and colonic organoid systems to study the effect of *Pink1* on epithelial activity in an isolated system.

**Methods:**

Single-cell RNA sequencing was performed on colonic IECs isolated from *Pink1* WT and KO mice, at steady state and following *in vivo C. rodentium* infection. Mice were sacrificed at D6 post infection to elucidate transcriptional differences between IEC lineages of each genotype. To validate scRNAseq findings, we derived *ex vivo* colonoids from *Pink1* WT and KO mouse crypts and used a *Pink1* KO CMT-93 epithelial cell line with WT clone as control. The cultures were treated with lipopolysaccharide (LPS), or infected with gram-negative pathogens to determine how PINK1 loss-of-function affects the inflammatory response of the epithelium.

**Results:**

At baseline, our data revealed upregulation of interferon (IFN)-signaling genes (ISGs) in *Pink1* KO enterocytes (ECs) compared to WT; whereby more than 70% of upregulated differentially expressed genes were linked to type I or II IFN signaling. *In vitro* stimulation of *Pink1* KO epithelial cell line with IFN resulted in stark upregulation of ISGs compared to WT. During infection, *Pink1* KO ECs demonstrated significant alterations in actin-cytoskeleton-related GO term pathways compared to WT. Infection of *Pink1* KO CMT-93 epithelial cells revealed morphological changes in F-actin structures.

**Conclusions:**

Using *in vivo* and *in vitro* models encompassing PD-genetic susceptibility and environmental stimuli, we identified dysregulation in *Pink1* KO epithelium, suggesting altered inflammatory responses at baseline and infection. By investigating PD-associated genes in IECs, we will contribute to better understanding the role of the intestine in PD initiation, progression, and pathogenesis.

**Funding Agencies:**

CAG, CIHR

